# Comprehensive quantitative analysis of alternative splicing variants reveals the *HNF1B* mRNA splicing pattern in various tumour and non-tumour tissues

**DOI:** 10.1038/s41598-021-03989-z

**Published:** 2022-01-07

**Authors:** Jan Hojny, Romana Michalkova, Eva Krkavcova, Quang Hiep Bui, Michaela Bartu, Kristyna Nemejcova, Marta Kalousova, Petra Kleiblova, Pavel Dundr, Ivana Struzinska

**Affiliations:** 1 Institute of Pathology, First Faculty of Medicine, Charles University and General University Hospital in Prague, Studničkova 2, 12800 Prague 2, Czech Republic; 2 Institute of Medical Biochemistry and Laboratory Diagnostics, First Faculty of Medicine, Charles University and General University Hospital in Prague, Kateřinská 32, 121 08 Prague 2, Czech Republic; 3 Institute of Biology and Medical Genetics, First Faculty of Medicine, Charles University and General University Hospital in Prague, Albertov 4, 128 00 Prague 2, Czech Republic

**Keywords:** Cancer genetics, Gene expression, RNA sequencing, Reverse transcription polymerase chain reaction, RNA metabolism, Alternative splicing

## Abstract

Hepatocyte nuclear factor-1-beta (HNF1B) is a transcription factor and putative biomarker of solid tumours. Recently, we have revealed a variety of *HNF1B* mRNA alternative splicing variants (ASVs) with unknown, but potentially regulatory, functions. The aim of our work was to quantify the most common variants and compare their expression in tumour and non-tumour tissues of the large intestine, prostate, and kidney. The *HNF1B* mRNA variants 3p, Δ7, Δ7–8, and Δ8 were expressed across all the analysed tissues in 28.2–33.5%, 1.5–2%, 0.8–1.7%, and 2.3–6.9% of overall *HNF1B* mRNA expression, respectively, and occurred individually or in combination. The quantitative changes of ASVs between tumour and non-tumour tissue were observed for the large intestine (3p, Δ7–8), prostate (3p), and kidney samples (Δ7). Decreased expression of the overall *HNF1B* mRNA in the large intestine and prostate cancer samples compared with the corresponding non-tumour samples was observed (p = 0.019 and p = 0.047, respectively). The decreased mRNA expression correlated with decreased protein expression in large intestine carcinomas (p < 0.001). The qualitative and quantitative pattern of the ASVs studied by droplet digital PCR was confirmed by next-generation sequencing, which suggests the significance of the NGS approach for further massive evaluation of the splicing patterns in a variety of genes.

## Introduction

Hepatocyte nuclear factor-1-beta (HNF1B, also known as Transcription Factor-2, TCF2; MIM#189907) is a transcription factor which plays an important role in the regulation of the development of a number of tissues and organs during embryogenesis^1–4^. Apart from its role in differentiation, the HNF1B protein also regulates the expression of multiple genes involved in cell cycle modulation, susceptibility to apoptosis, and response to oxidative stress^5,6^. Its expression in adults was detected in tubule forming epithelial tissues such as kidney and pancreatic exocrine duct tubules, the colon, small intestine, stomach, testes, lungs, liver, prostate etc. (GTEx Portal)^7^. In recent years, a number of studies have suggested that HNF1B may be involved in the tumorigenesis of several types of solid tumours, such as clear cell carcinoma of the ovary, renal cell carcinoma of the kidney, tumours of the gastrointestinal tract, the liver, the pancreas, and the prostate^6,8–14^.

Our previous work showed a loss of the HNF1B protein expression in several solid tumours, and thus suggests that HNF1B may act in a tumour suppressive fashion in colorectal carcinoma (CRC)^15^, clear cell renal carcinoma (ccRCC) and chromophobe renal cell carcinoma^16^, prostate carcinoma (PC)^17^, and high-grade serous carcinoma (HGSC)^18^. The *HNF1B* gene promoter methylation (as a typical factor leading to a loss of protein expression) was observed in 55% PC and 38% HGSC samples. Tumour samples from CRC and ccRCC showed sporadic methylation of the *HNF1B* promoter, as well as the presence of truncating mutations in the *HNF1B* gene.

**Figure 1:**
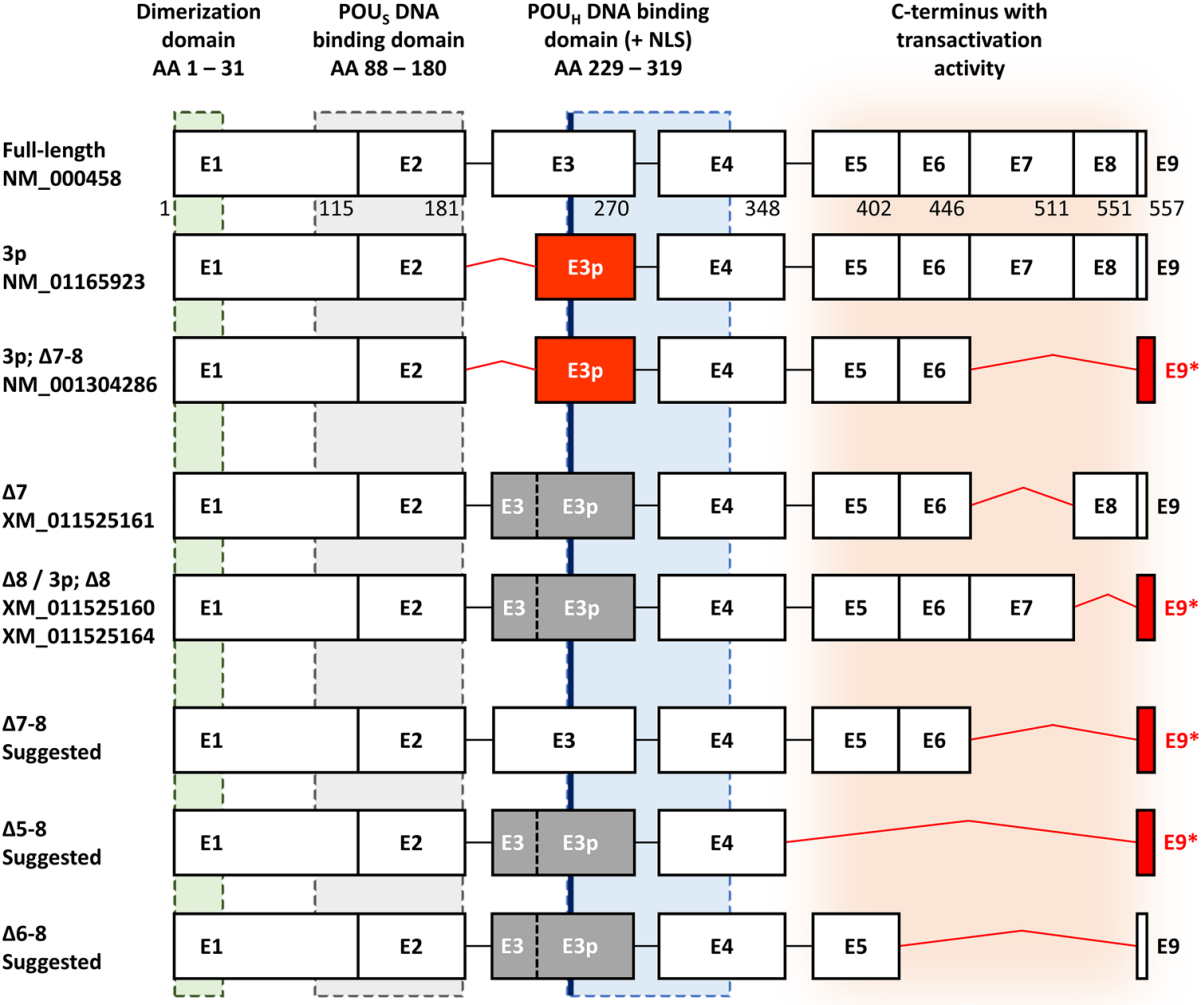
Scheme of the proposed *HNF1B* splicing pattern. White boxes represent canonical exons, black lines represent canonical exon-exon junctions. Red boxes represent alternatively spliced exon (E3p) or exon with alternative stop codon (E9*), red lines represent confirmed alternative exon-exon junctions. Gray boxes represent undetermined variant of exon 3 in suggested transcripts. The lengths of the exons are proportional. Amino acid (AA) numbers are indicated below the exon boxes of full-length transcript. The green, grey, blue, and orange areas illustrate the coding areas for functional domains across the *HNF1B* transcripts. *NLS* nuclear localization signal (thick blue line). *POU*_*S*_ POU specific domain. *POU*_*H*_ POU homeodomain. The scheme of *HNF1B* transcripts is based on previous findings^19^ and actual RefSeq database information.

As a candidate regulatory mechanism of the *HNF1B* gene expression, and due to the incomplete and often contradictory information about *HNF1B* ASVs spectrum, we recently thoroughly investigated the presence and distribution of *HNF1B* mRNA alternative splicing variants (ASVs). The *HNF1B* reference transcript comprises nine coding exons and produces full length 557 amino acid protein (RefSeq NM_000458). In our previous work, we performed mainly qualitative analyses and described 45 splicing events in a limited number of samples (eight representative samples per analysed tissue pool)^19^. By this approach we have identified predominant and predominant-candidate *HNF1B* ASVs (Fig. 1), which included alternatively spliced exon 3p (exon 3 which lacks 78 bp at 5′; known as variant NM_01165923.4), Δ7 (deletion/intronization of exon 7; predicted in XM_011525161.1), Δ7–8 (known in transcript NM_001304286.2 where was described in combination with 3p variant), Δ8 (predicted in XM_011525164.1 and XM_011525160.1), Δ5–8 and Δ6–8 (both not previously described in databases). All the named variants maintain an open reading frame or contain an alternative (not premature) stop codon. Corresponding protein products were detected and described for 3p and Δ7–8 ASVs (NP_001159395.1 and NP_001291215.1, respectively). So far, quantification of ASVs and their co-occurrence in *HNF1B* transcripts have not been fully elucidated. Therefore, *HNF1B* ASVs 3p, Δ7, Δ7–8, Δ5–8, and Δ6–8 ASVs were chosen for subsequent precise quantitative characterization in a wide spectrum of tumour (T) and non-tumour (NT) samples in a number of tissues presented in this work.

The aim of our study was (i) to evaluate the expression of *HNF1B* ASVs and their co-occurrence in the final transcripts in a spectrum of NT samples including the pancreas, large intestine, prostate, kidney and female internal genital tract, and thus to complete the *HNF1B* splicing pattern in non-malignant samples; (ii) to evaluate *HNF1B* overall expression and to quantify the proportion of *HNF1B* ASVs expressed in T sample sets including CRC, ccRCC, PC, and pancreatic carcinoma and their NT counterparts; (iii) to show the usability of the capture RNA next-generation sequencing (RNA-Seq) approach for the detection and quantification of ASVs. The precise characterization of the *HNF1B* splicing pattern in T and NT tissues may contribute to a better general understanding of the *HNF1B* gene expression and the role of *HNF1B* ASVs in tumorigenesis.

## Results

### Expression of ASVs 3p, Δ7, Δ7–8, and Δ8 was detected in all analysed NT tissue samples

The droplet digital PCR (ddPCR) analysis of NT samples revealed the expression of the 3p, Δ7, Δ7–8, and Δ8 ASVs in all 146 NT samples in the subset of samples with sufficient *HNF1B* mRNA expression (see methods). The proportion of *HNF1B* ASVs is similar among analysed tissue sets (Table 1), although the overall *HNF1B* expression level differs in different tissues^7^.

**Table 1:** The proportion of alternative splicing variants in selected non-tumour tissues.

ASV	Female internal genital tract NT (N = 31) (%)	Kidney NT (N = 31) (%)	Pancreas NT (N = 7) (%)	Prostate NT (N = 35) (%)	Large intestine NT (N = 42) (%)
3p	29.7	28.2	30.2	29.1	33.5
Δ7	1.9	1.7	1.9	1.5	1.7
Δ7–8	1.1	1.0	1.7	0.8	1.2
Δ8	3.2	3.8	6.9	2.3	6.7

Each value represents the percentage of the overall *HNF1B* mRNA expression (100%; calculated as the sum of canonical exon 3 and 3p variants). *N* number of samples, *NT* non-tumour tissue.

Variants Δ5–8 and Δ6–8 were detected during the initial analysis steps only in a portion of NT kidney samples (15/48 T and 31/48 NT). The measured expression levels were very low and close to the detection limit of the method used (< 0.5% of overall *HNF1B* expression). Considering the highest overall *HNF1B* mRNA expression in the NT kidney tissue compared to other tissues^7^, these variants were not analysed in other sample sets, and we considered them as minor *HNF1B* ASVs.

### ASVs Δ7, Δ7–8 or Δ8 exist also in combination with the ASV 3p in *HNF1B* transcripts

In a representative pool of NT kidney samples (see methods) PCR analysis of the *HNF1B* transcripts containing Δ7, Δ7–8 or Δ8 ASV was performed. In the PCR reactions unique primers for each ASV were used together with forward primer in exon 2, which allowed us to determine the variant of exon 3. The analysis confirmed the existence of transcripts containing each of these ASVs separately (with canonical exon 3), as well as in combination with the 3p ASV (Fig. S1).

### Reduced overall *HNF1B* mRNA expression was detected in colorectal and prostate cancer and correlates with low protein expression in colorectal cancer

**Figure 2:**
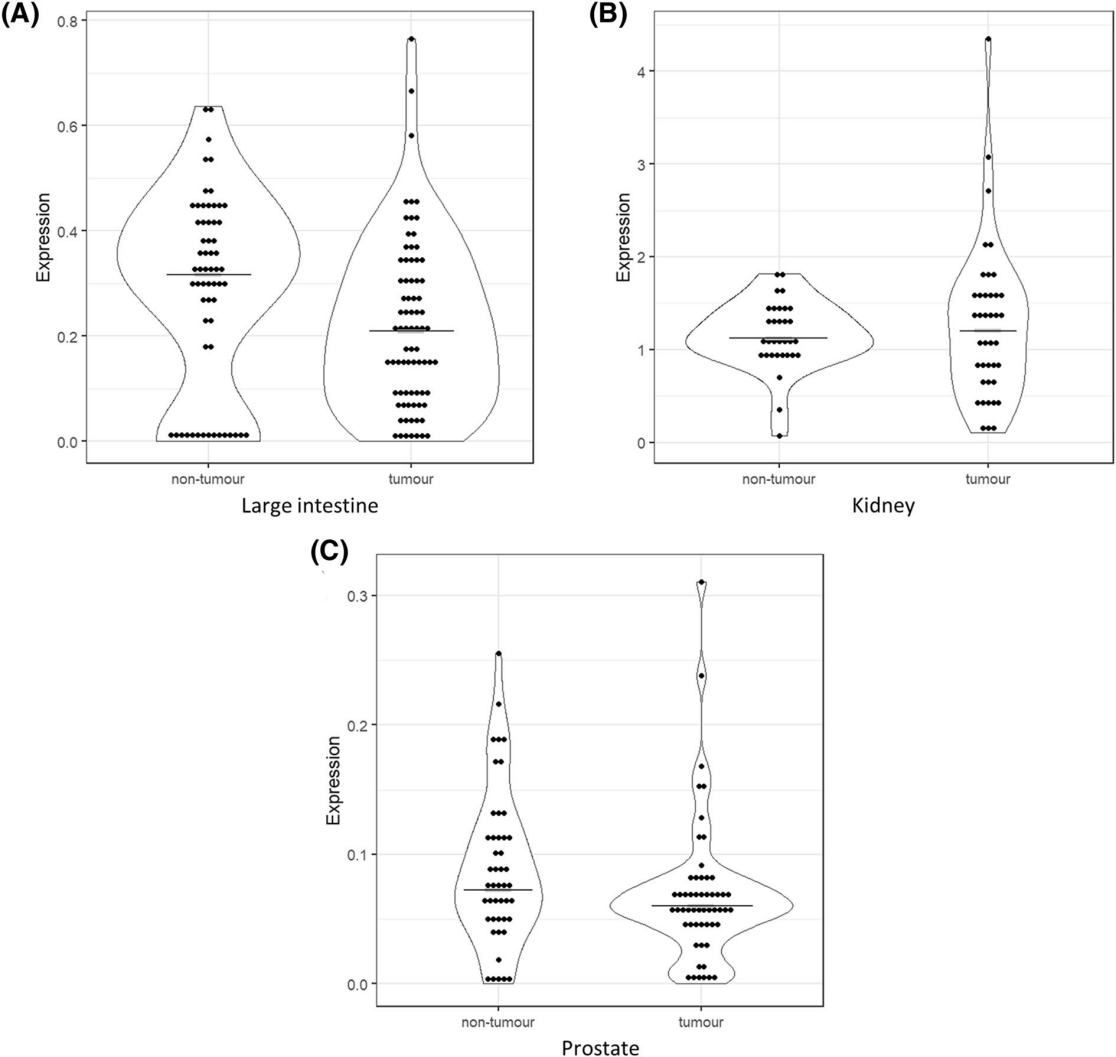
*HNF1B* mRNA overall expression levels in the tumour and non-tumour samples. (**A**) large intestine (NT = 61 samples; T = 78 samples; p = 0.019); (**B**) kidney (NT = 32; T = 40; p = 0.968); (**C**) prostate tissue (NT = 46; T = 55; p = 0.047). Data is visualized as violin plots. Each dot represents one sample. Expression is relative to *POLR2A* (*POLR2A* expression = 1). Black line represents median.

A comparison of the overall *HNF1B* expression between T and NT tissue samples was successfully performed in 78 T and 61 NT large intestine tissue samples, 40 T and 32 NT kidney samples, and 55 T and 46 NT prostate samples. A decreased overall expression of *HNF1B* was observed in the large intestine T samples in comparison to the NT counterparts (p = 0.019; Fig. 2A), and in prostate T samples in comparison to the NT samples (p = 0.047, Fig. 2C). There were no statistically significant differences in the expression observed in the T and NT kidney samples (Fig. 2B).

Paired comparisons of the overall *HNF1B* expression within the subsets of matched T and NT samples were performed in 61 large intestine tissue samples, 31 kidney tissue samples, and 46 prostate tissue samples. This statistical approach showed the same results compared to non-paired testing. The complete results are listed in Table 2A.

**Table 2:**
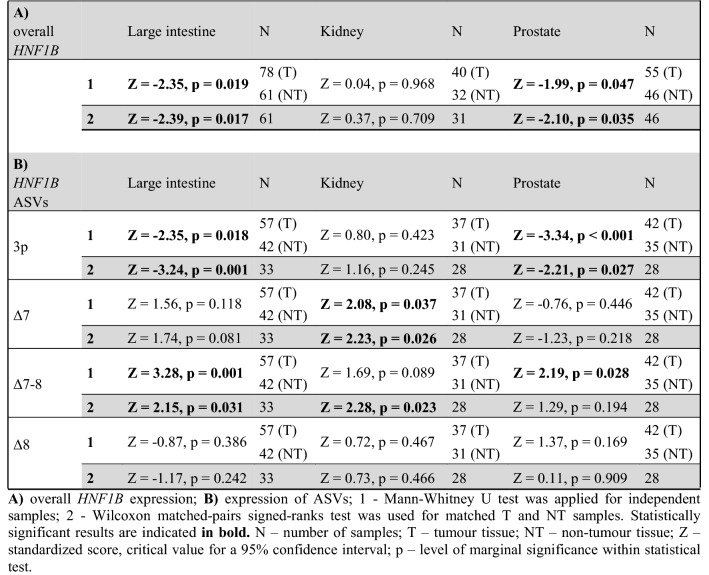
Comparison of overall expression or individual ASVs’ expression between tumour and non-tumour samples in different tissues.

(A) overall *HNF1B* expression; (B) expression of ASVs; 1—Mann–Whitney *U* test was applied for independent samples; 2—Wilcoxon matched-pairs signed-ranks test was used for matched T and NT samples. Statistically significant results are indicated in bold. *N* number of samples; *T* tumour tissue, *NT* non-tumour tissue, *Z* standardized score, critical value for a 95% confidence interval, *p* level of marginal significance within statistical test.

**Figure 3:**
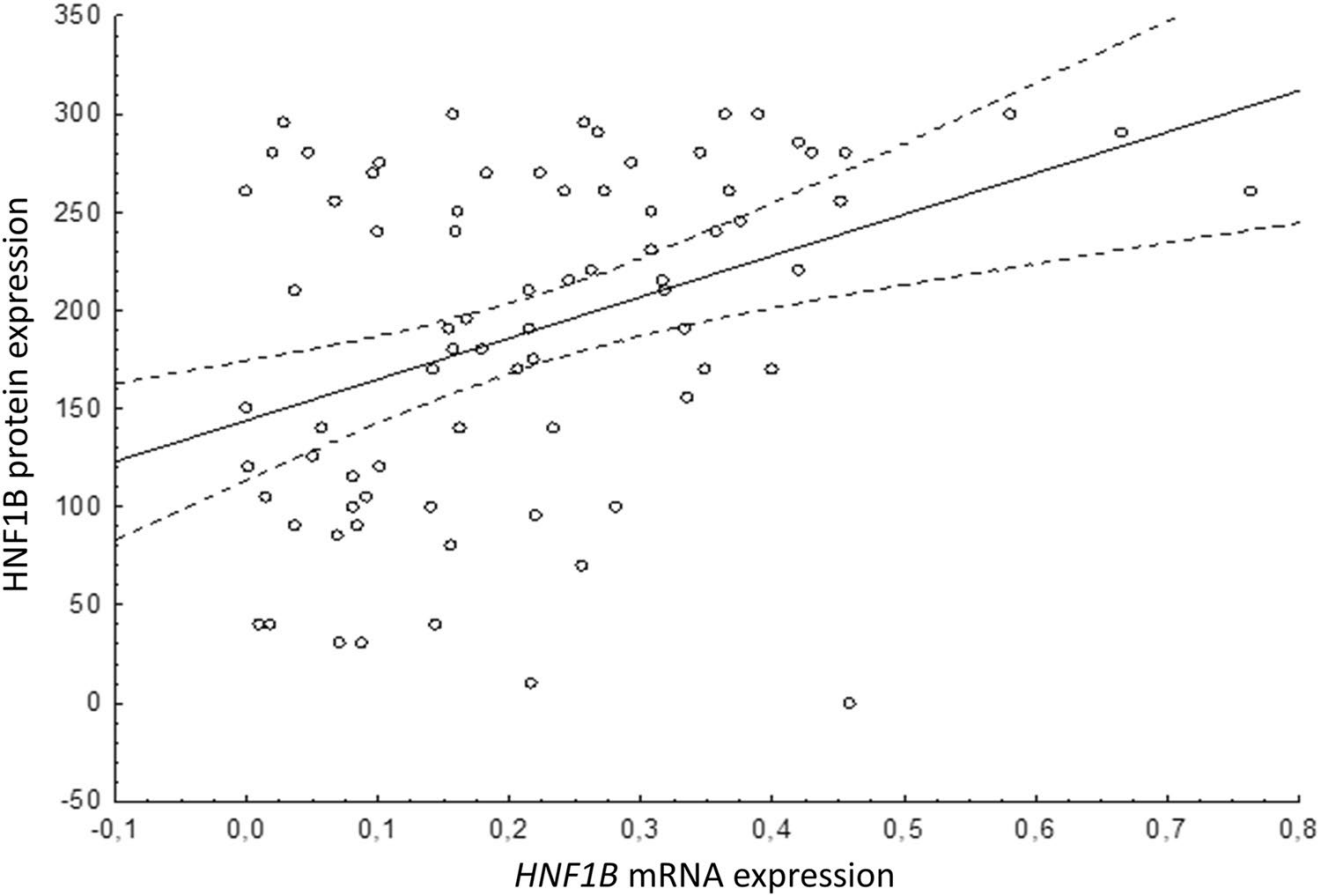
Correlation of the overall *HNF1B* mRNA and protein expression in 78 large intestine carcinoma samples showed weak positive correlation (R = 0.39; p < 0.001). *HNF1B* mRNA expression (X axis) is relative to *POLR2A* (*POLR2A* expression = 1). HNF1B protein expression (Y axis) is calculated as H-score. Each dot represents one sample. Dashed lines indicate the 95% confidence intervals for the regression line.

We further correlated the mRNA expression with protein expression in 78 CRC. The immunohistochemical protein expression (H-score) of the same CRC sample set was assessed previously by our group^15^. The comparison revealed a weak positive correlation (R = 0.39, F = 13.8, df = 1.76, p < 0.001; Fig. 3). A similar analysis was published previously for the same sample set of prostate carcinomas, where no correlation between mRNA and protein expression was observed^17^.

### Differences between the relative expression of the *HNF1B* ASVs in tumour and non-tumour samples

**Figure 4:**
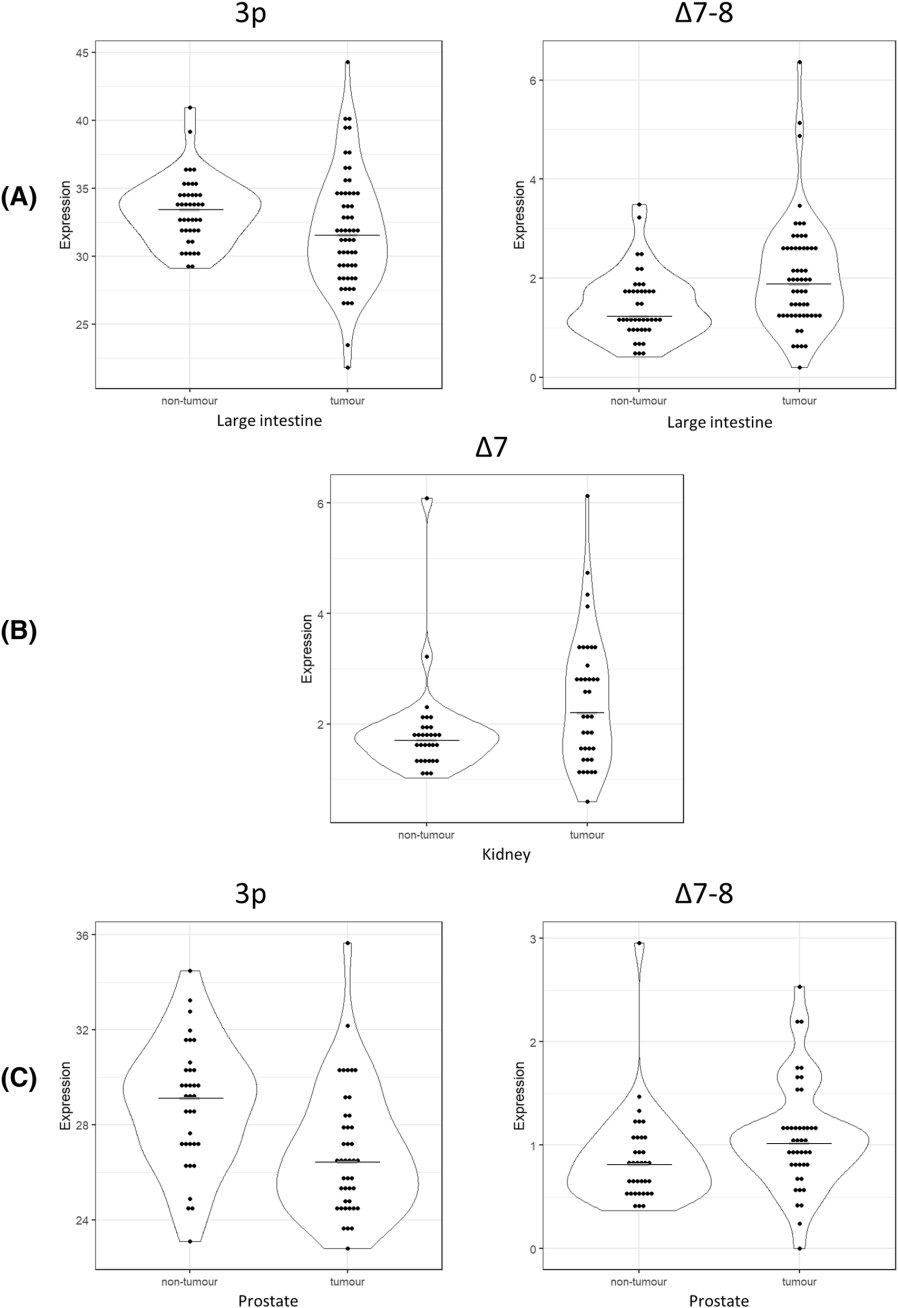
Significantly different expression levels of the *HNF1B* alternative splicing variants in T and NT tissue samples. (**A**) Large intestine (NT = 42 samples; T = 57 samples); (**B**) kidney (NT = 31 samples; T = 37 samples); (**C**) prostate (NT = 35 samples; T = 42 samples). Data is visualized as violin plots. Each dot represents one sample. Expression is relative to overall *HNF1B* mRNA expression (100). Black line represents median. Complete results are visualized in Fig. S2.

The analysis of *HNF1B* ASVs expression was successfully performed in the subset of samples with sufficient *HNF1B* expression (see methods). The statistical evaluation was performed in 57 T and 42 NT tissue samples of large intestine, 37 T and 31 NT kidney samples, and 42 T and 35 NT prostate samples. The relative expression of the 3p variant was significantly decreased in T compared with NT tissues of the large intestine samples (median T = 31.6%; NT = 33.5%; p = 0.018; Fig. 4A) and the prostate samples (median T = 26.5%; NT = 29.1%; p < 0.001; Fig. 4C). On the contrary, an increased expression of the Δ7 variant was detected in the kidney tissue T samples when compared with the kidney NT samples (median T = 2.2%; NT = 1.7%; p = 0.037, Fig. 4B). An increased expression of the Δ7–8 variant was detected in T samples in comparison to NT samples in the prostate tissue (median T = 1.0%; NT = 0.8%; p = 0.028, Fig. 4C), and the large intestine samples (median T = 1.9%; NT = 1.2%; p = 0.001, Fig. 4A). The expression of the alternative variant Δ8 did not show any statistically significant differences in the tissues analysed. The complete results are provided in Table 2B.

Paired comparisons of ASVs within the matched T and NT samples were performed in 33 large intestine tissue samples, 28 prostate tissue samples, and 28 kidney tissue samples. In accordance with the non-paired testing, significant differences were observed for the 3p ASV in the large intestine and prostate paired samples, the Δ7 ASV in the kidney paired samples, and the Δ7–8 ASV in the large intestine. Although the comparison between statistical approaches of individual ASVs showed the same trend, no statistically significant differences were observed in the case of Δ7–8 ASV in the prostate and kidney paired samples. The complete results are provided in Table 2B and Fig. S2.

The *HNF1B* DNA mutation analysis of this sample set was done and published previously^15–17^ and did not reveal any variant located in canonical or potential cryptic splice site of T or NT samples in the coding *HNF1B* regions with flanking intronic sequences (+ − 20 bp). Overall, few different variants were detected in our dataset. All these variants were tested for potential splice effect by in-silico tools with any potential splice predictions. Moreover, we did not detect any major expression deviations of analysed ASVs in our dataset which could potentially indicate aberrant splicing resulted from the variant located in these sites. Although we cannot exclude the potential splicing effect of deep intronic variants, our findings suggest that all analysed splicing variants were created based on the alternative splicing mechanism and described differences in ASVs expression between T and NT sample sets are most probably resulting from the changes in alternative splicing.

### Capture RNA-Seq confirmed the *HNF1B* quantitative and qualitative splicing pattern determined by ddPCR

**Figure 5:**
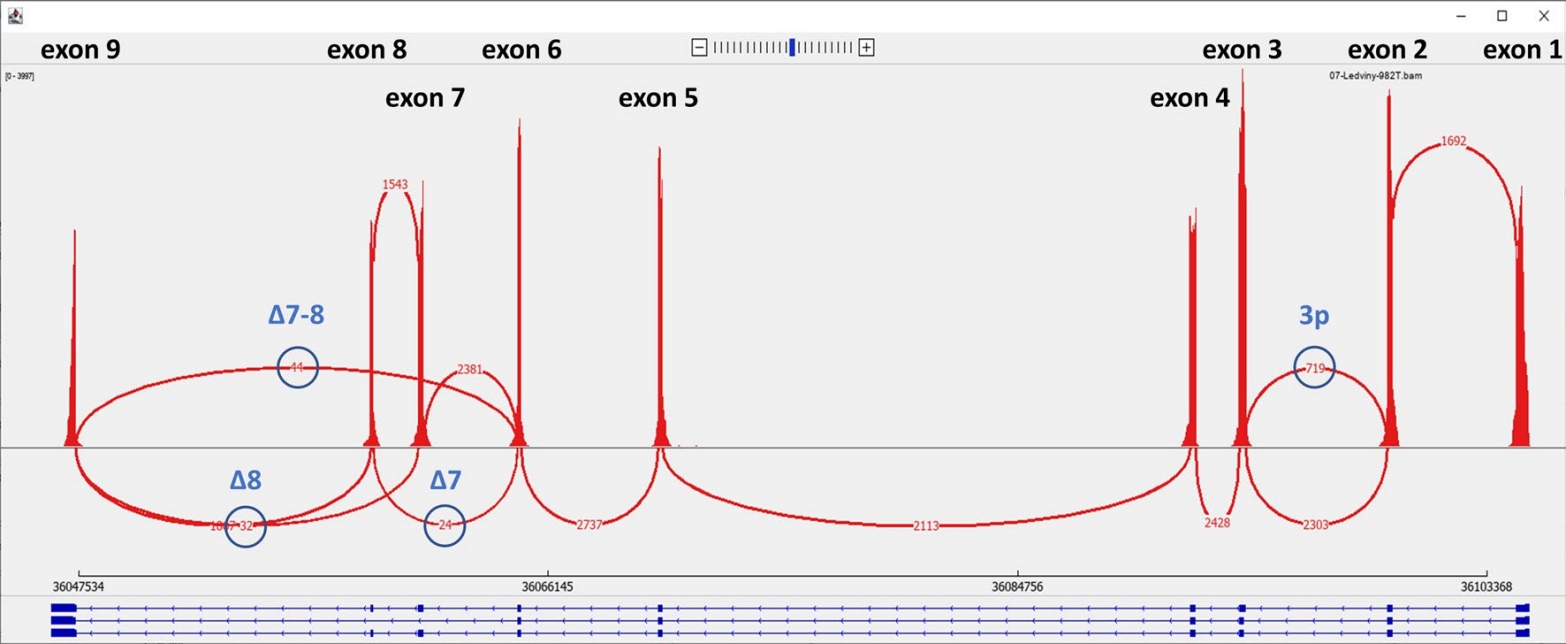
Representative example of the *HNF1B* splicing pattern based on the capture RNA-Seq of a kidney T sample visualised as a sashimi plot in IGV (Broad Institute). The height of the peaks shows exon coverage, the red lines show splicing reads, the red numbers in the red lines show the number of splicing reads, the ASVs are indicated in blue font and the number of ASVs’ reads is circled. The dark blue scheme under the plot shows the gene structure of the protein coding *HNF1B* transcripts (blue boxes indicate exons; thin horizontal lines indicate introns). The cut-off for visualisation of the splicing reads was set to 10. The results are provided in Table 3.

The RNA from six selected paired tissue samples with detected differences in the splicing patterns between T and NT were sequenced by the capture RNA-Seq approach. A representative example of one kidney T sample is visualised in Fig. 5. In all 12 representative samples reads were identified which corresponded to 3p, Δ7, Δ7–8, and Δ8 ASVs. Normalized NGS data (ratio of individual splicing reads) showed a similar relative expression of the studied variants when compared to ddPCR quantitative analysis (Table 3). Moreover, we did not identify any novel *HNF1B* splicing variants by capture RNA-Seq other than those reported in our previously published work, where deep sequencing of individual *HNF1B* exon-exon junctions was used, which could potentially miss the ASVs^19^.

**Table 3:** The proportion of alternative splicing variants calculated from ddPCR-based and NGS-based data in selected paired tumour and non-tumour tissues (with raw data overview).

	3p		Δ7		Δ7–8		Δ8		ddPCR	NGS
	ddPCR (%)	NGS (%)	ddPCR (%)	NGS (%)	ddPCR (%)	NGS (%)	ddPCR (%)	NGS (%)	tmp/µl	cov
Pancreas T	29.9	27.5	2.8	1.5	2.6	3.2	10.4	5.7	385	3766
Pancreas NT	30.2	30.5	3.9	1.7	2.8	4.2	11.4	5.2	165	1409
Large intestine T	33.2	32.8	3.9	3.7	5.1	5.6	15.1	6.9	214	2572
Large intestine NT	32.7	32.5	1.8	0.7	1.2	1.2	6.0	3.9	281	2775
Prostate T	24.4	21.2	4.4	1.8	2.2	2.5	4.8	2.5	66	663
Prostate NT	29.7	27.2	1.2	1.8	0.5	0.4	2.7	1.0	158	2490
Kidney T	30.1	23.8	4.7	0.8	2.7	1.5	6.9	1.1	916	3897
Kidney NT	28.4	25.2	1.2	1.1	0.6	1.0	2.3	1.9	981	11,619
Endometrium T	27.4	25.9	0.4	0.1	0.8	0.7	1.5	0.3	170	2301
Endometrium NT	31.5	24.4	1.6	0.8	0.8	0.9	2.7	2.3	203	1786
Ovary T	19.8	16.4	1.8	2.4	2.1	2.0	9.1	3.3	137	866
Ovary NT	30.0	24.3	2.0	1.2	1.1	2.0	5.2	3.5	113	1082

Numbers represent the percentage of each individual variant related to the overall *HNF1B* mRNA expression (calculated as the sum of canonical exon 3 and 3p variants in both approaches). Each line represents data from one representative sample; ddPCR tmp/µl represents number of *HNF1B* templates per 1 µl of cDNA detected by ddPCR; NGS cov. represents peak coverage of *HNF1B* exon 3. *T* tumour tissue, *NT* non-tumour tissue.

### TCGA SpliceSeq database data show similar expression of analysed *HNF1B* ASVs

For further validation of our results, we used RNA-Seq based data from TCGA SpliceSeq database^20^ (TCGAss) and compared them with our ddPCR expression results. Although the TCGAss data are limited due to the whole transcriptome sequencing with rather lower sequence depth which was not designed for thorough quantitative analysis of low expressed alternative transcripts in genes such as *HNF1B*, we were able to directly assess the expression of variants 3p, Δ7–8 and Δ8 in paired T and NT datasets that correspond to ours (Table 4). Variant Δ7 as a simple exon skipping variant was absent in the downloadable TCGAss data probably due to the low coverage/number of supporting reads.

**Table 4:**
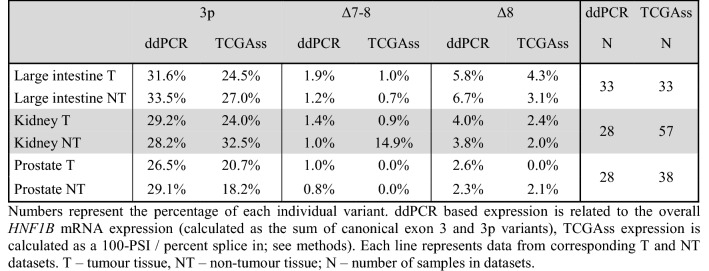
Comparison of *HNF1B* ASVs expression measured by our ddPCR approach with TCGAss.

Numbers represent the percentage of each individual variant. ddPCR based expression is related to the overall *HNF1B* mRNA expression (calculated as the sum of canonical exon 3 and 3p variants), TCGAss expression is calculated as a 100-PSI/percent splice in; see methods). Each line represents data from corresponding T and NT datasets. *T* tumour tissue, *NT* non-tumour tissue, *N* number of samples in datasets.

Comparison shows similar expression levels, usually in units of percent, in most cases with several exceptions. Most distinctive exception was Δ7–8 expression in kidney NT tissue with median of 14.9% (compared to 1% measured by ddPCR) and 3p expression in prostate NT tissue with median 18.2% (compared to 29.1% measured by ddPCR).

## Discussion

Although the significance of HNF1B in tumorigenesis has recently been closely analysed and discussed, its exact role and mechanism of action have not yet been fully clarified. There are studies suggesting that HNF1B may act as an oncogene in specific cancer types, such as ovarian clear cell carcinoma^5,6,21^ and papillary renal cell carcinoma^16^. Other studies suggested that HNF1B acts mainly as a tumour suppressor in colorectal, prostate, ovarian, and some other types of solid tumours^3,15,17,18,22^. One of the possible explanations for this ambivalent character is the existence of one or more alternative splicing variants with dysregulated expression in tumour tissues which have either a regulatory role or code for HNF1B protein isoforms with different function^19^.

Our previous work showed that the splicing mechanism in ovarian, colorectal, kidney, pancreatic, and prostate tissue types can create a wide range of *HNF1B* alternative splicing events. Out of the 45 previously described ASVs of *HNF1B* mRNA, variants 3p, Δ7, Δ7–8, and Δ8 occurred in all analysed tissue pools in a higher portion, and variants Δ5–8 and Δ6–8 occurred in a majority of the tested tissue pools in moderate portion^19^. Moreover, according to our previous findings and the RefSeq database information (accessed April 30, 2021), all these ASVs have fully defined open reading frame and thus they potentially code for protein products (Fig. 1)^19^. Therefore, these ASVs were selected for further investigation by a precise ddPCR quantitative approach on a large-scale tumour and non-tumour sample set presented in this study. We confirmed the previous assumption that variants 3p, Δ7, Δ7–8, and Δ8 are expressed ubiquitously. These variants were detected in all 146 analysed NT tissue samples. The variant 3p corresponded to approximately 30% of all *HNF1B* transcripts, ASV Δ8 was detected in 2–7% of all *HNF1B* transcripts, and variants Δ7 and Δ7–8 were detected in relatively low levels in approximately 1–2% of all *HNF1B* transcripts (Table 1). On the other hand, the previously described variants Δ5–8 and Δ6–8 were identified only in a portion of the tested samples and showed expression levels lower than 0.5% of the overall *HNF1B* mRNA expression, which is in accordance with our previous ASVs overview^19^ and emphasizes the rather lower significance of these *HNF1B* ASVs.

Moreover, the TCGAss database information^20^, which is based on the TCGA RNA-Seq data (https://bioinformatics.mdanderson.org/TCGASpliceSeq), supports the presence of all four described variants in the majority of other tumour tissues if the expression filter for minor splice variants in the database (Min minor splice expression) is decreased from default 10% to 1%.

Further analysis of the alternative *HNF1B* transcripts revealed that the ASVs Δ7, Δ7–8, and Δ8 occur in alternative transcripts separately or in combination with ASV 3p. This information completes the look at the *HNF1B* splicing pattern and supports the existence of predicted *HNF1B* transcripts containing full-length exon 3 and Δ7 variant (XM_01525161.1); full-length exon 3 and Δ8 variants (XM_011525164.1); alternative 3p and Δ8 variants (XM_011525160.1), and further suggests the existence of full-length exon 3 and Δ7–8 variants (novel) and alternative exon 3p and Δ7 variants (novel).

Other predicted *HNF1B* transcripts XM_011525162.2 and XM_011525163.2 (which contain canonical exons 1–4 and are concluded by the exonization of a part of intron 4) were not detected in any samples analysed by the NGS approach. This suggests that these transcripts are either expressed in low levels (< 1%) and thus under the detection limit of the used NGS method, or specific for a type of tissue which was not analysed in our study.

Previous *HNF1B* DNA mutation analysis of presented sample set^15–17^ did not reveal any variant located in canonical or potential cryptic splice sites nor the variant with potential splice effect in coding sequence and exon flanking areas of the *HNF1B* gene. Even though potential variants with splice effect located deep in the introns were not analysed, based on even expression pattern of ASVs, we can conclude, that detected frequencies of analysed *HNF1B* ASVs are based on the alternative splicing rather than aberrant splicing mechanism.

So far, only two studies have analysed the expression of *HNF1B* ASVs. Specifically, they compared only the canonical variant [i.e., full-length *HNF1B* transcript, named by the authors as *HNF1B*(A)] and the alternative transcript 3p variant [named by the authors as *HNF1B*(B)]^23,24^. Our results are not in full accordance with those authors. When they compared the 3p variant to the full-length *HNF1B* mRNA (by qPCR normalization to reference B2M mRNA expression), they observed approximately 60% lower levels of the 3p ASV in the pancreatic islets, a balanced expression in the kidney tissues, and approximately 50% higher expression in the pancreas^23^. Based on our ddPCR approach we found that the median expression of ASV 3p is about 50% lower than the expression of the full-length exon 3 variant in all 142 analysed NT tissues (Table 1). In their later study, Harries et al*.* showed that the expression of the full-length *HNF1B* transcript is non-significantly slightly lower (approximately 0.8 ×), and the expression of the 3p variant is significantly higher (approximately 7 ×) in 21 prostate adenocarcinoma tissue samples when compared with 39 non-malignant (benign hyperplasia) tissues (by qPCR normalization to B2M and GUSB reference mRNA transcripts)^24^. Based on our current study, we can state that the overall *HNF1B* expression is similarly lower (approximately 0.9 ×) in prostate T samples compared with NT samples with the marginal statistical signification (Fig. 2C, Table 2A). In contrast to the results published by Harries et al*.*, our data showed that the expression of the 3p variant is significantly lower in prostate adenocarcinomas when compared with prostate NT samples (Fig. 4C, Table 2B). However, it cannot be reliably macroscopically distinguished between benign hyperplasia and non-benign character of samples in our NT prostate sample group even though all tissue specimens were evaluated by the trained pathologists. Although we do not expect major differences of the *HNF1B* ASVs expression in healthy prostate tissue samples compared to benign hyperplasia prostate tissue samples and our data from individual NT samples did not deviate, our results may be affected by this issue.

To our knowledge, neither *HNF1B* overall expression in other tissue types nor the expression of individual alternative transcripts has been previously analysed or published by other authors. However, we were able to compare our data to TCGAss database (Table 4) which provides transcript splicing patterns on the base of The Cancer Genome Atlas project (TCGA) RNA-Seq datasets. Direct comparison of the *HNF1B* ASVs expression results showed certain level of similarity, despite the different approaches for sample analysis and ASVs frequency calculation.

Unfortunately, TCGA RNA-Seq sequencing depth was not sufficient for the unbiased statistical evaluation of the *HNF1B* ASVs expression in T versus NT tissue samples and its comparison to our results obtained by sensitive ddPCR results. The TCGAss data showed approx. 4 × lower coverage depth in exon-exon junctions in comparison with our panel RNA-Seq (Table 3). We consider this as insufficient for the reliable low-expressed ASVs detection which is reflected at Table 4. Expression of variant Δ7 was not evaluated by TCGAss in individual samples (see methods), thus it is not reported there and variants Δ7–8 and Δ8 showed minimal expression in prostate T and NT sets, where is the overall *HNF1B* expression lowest, which is most likely caused by insufficient sensitivity of used method. These limitations could also be the reason for the observed differences in Table 4.

Our data revealed that the only tissue with a strong significant difference of the overall *HNF1B* expression when comparing NT with T samples was the large intestine (Fig. 2A). Our analysis showed that the significantly lower overall *HNF1B* mRNA expression in the large intestine carcinoma samples correlates with low protein expression in the same sample set (Fig. 3)^15^. Given the previous results gained from the same set of large intestine carcinoma samples which showed that the effect of the *HNF1B* gene promoter methylation and the *HNF1B* mutation is minor^15^, we can propose that the decreased *HNF1B* mRNA expression in the large intestine carcinoma samples is most probably caused by a reduction of the *HNF1B* transcriptional rate, which is usually directly affected by upstream gene expression regulators.

Another important mechanism of the potential reduction of the overall HNF1B protein function could be the effect of alternative splicing. A significant increase in the expression of the Δ7–8 variant was observed in the large intestine T samples when compared with the NT samples, by both statistical approaches (Fig. 4A,C; Table 2B). In the kidney T samples, a significant increase of the Δ7 variant expression was detected compared with the kidney NT samples (Fig. 4B; Table 2B). The transcript Δ7–8, as well as the Δ7 or Δ8 variants, lacks the part which codes an important domain responsible for the transcriptional activation of *HNF1B* gene targets. The same DNA binding capacity but lower transactivation activity of the proteins raised from the *HNF1B* Δ7–8/Δ7/Δ8 transcripts could be expected, but a functional analysis of these predicted proteins is needed prior to the final evaluation of their role and effect.

Interestingly, the variant 3p, which lacks 78 bp at its 5′ end of exon 3 (which codes for a spacer between the first and the second DNA binding domain of the HNF1B protein) shows a significantly lower expression in the large intestine T samples when compared with the NT samples (Fig. 4A). A similar observation was made in the prostate T samples, where a significantly lower expression was also detected compared with the NT samples (Fig. 4C; Table 2B). Regarding the high expression of the 3p ASV of all *HNF1B* transcripts, we can assume that this transcription variant codes for a protein with a similar transcriptional activation potential. However, as with other variants, a functional assay should be done prior to the final evaluation of the HNF1B 3p isoform protein function and its role in tumorigenesis.

Although we described statistically significant differences in the expression of certain ASVs in the T samples compared with the NT samples, the variances are relatively low in summary. Therefore, changes in the *HNF1B* splicing pattern are probably not the key mechanism in the regulation of gene expression of fully functional HNF1B protein in the analysed tumour tissues. Observed changes in the *HNF1B* splicing pattern may be a consequence of the overall tumour splicing disbalance rather than presence of potential deep intronic variants with splicing effect. However, this assumption should be confirmed by a suitable large-scale method such as RNA-Seq, where the multiple splicing patterns could be targeted and evaluated at once.

Finally, another interesting part of this work was the direct comparison of the *HNF1B* splicing data acquired by both ddPCR and capture RNA-Seq (Table 3). This comparison showed that a well-chosen normalization (in this case the sum of the canonical exon 3 and alternative exon 3p reads in NGS and expression in ddPCR) provides similar quantitative results for the variants 3p, Δ7, and Δ7–8 in a majority of the analysed tissue types. Quantification of the Δ8 variant showed greater variability across tissues, which was probably caused by non-ideal probes binding or NGS reads mapping in this area. Nevertheless, we believe that additional fine-tuning of the sample preparation and NGS data analysis can lead to a true quantitative analysis of the splicing pattern by the RNA capture NGS approach. Further, capture-based results in contrast with the whole transcriptome data usually benefits from higher coverage of low-expressed targeted genes, such as *HNF1B*. In comparison with external databases such as TCGAss or GTEx Portal^7^, we showed approx. 4 × greater coverage of the *HNF1B* exon-exon junctions which allows us to precisely calculate the expression even for the minor (< 10%) alternative transcripts. In addition to the other information gained by RNA NGS approach, analysis of the splicing pattern in a variety of genes can be beneficial. The capture RNA-Seq approach would bring increased throughput and decreased economic demands compared to the otherwise used large-scale quantitative splicing experiments.

In conclusion, our work unravels the spectrum of *HNF1B* alternative splicing. We described the spectrum of *HNF1B* ASVs in non-malignant tissues and the quantitative changes of the *HNF1B* ASVs in corresponding tumours. The exact function of individual ASVs is not fully clarified and should be thoroughly functionally determined. Our work provided a guide for the prioritization of alternative variants/isoforms for such experiments. Moreover, we described quantitative changes on the level of overall *HNF1B* mRNA expression and showed a positive correlation between decreased mRNA level and decreased protein level in colorectal cancer. Additionally, we showed that the NGS approach of capture RNA-Seq could be beneficial for the collective evaluation and direct quantification of the analysed splicing patterns.

## Materials and methods

### Patients and samples

Samples for the study were provided by The Bank of Biological Material, First Faculty of Medicine, Charles University. The tissue samples were collected during surgical procedures and processed by trained pathologists, including macroscopic evaluation of the whole resected tissue specimen prior to fixation. Representative tumour and paired non-tumour tissue samples (taken from the periphery of each resected specimen where available) were stored in RNAlater (Thermo Fisher) according to the manufacturer’s instructions until the genetic material had been isolated. The total amount of 512 tumour (T) and non-tumour (NT) tissue samples were obtained from the pancreas (14 adenocarcinomas; 9 NT), large intestine (83 adenocarcinomas; 82 NT), prostate (73 adenocarcinoma; 49 NT), kidney (59 clear cell renal cell adenocarcinomas; 43 NT), and female genital tract (100 NT). Non-tumour female genital tract samples were collected from the ovary, fallopian tubes, endometrium, and cervix; therefore, they are collectively referred to as “Female internal genital tract NT samples”. One high-grade serous carcinoma sample and one endometroid endometrial carcinoma sample was additionally included into the dataset due to the evaluation of NGS approach.

The study has been approved by The Ethics Committee of General University Hospital in Prague in compliance with the Helsinki Declaration (ethical approval number 41/16 as a part of the grant from the Czech Research Council 17-28404A) and all experiments were performed in accordance with these guidelines and regulations. The ethics committee which approved this retrospective study waived the need for informed consent.

### Total RNA isolation, quality control, and cDNA synthesis

The samples were processed according to the Digital MIQE Guidelines^25^ and as described before^17,19^. Briefly, the total RNAs and DNAs were isolated from the homogenized part of thawed tissue (10–30 mg) according to the Simultaneous Purification of Genomic DNA and Total RNA from Animal Tissues protocol by using an AllPrep DNA/RNA Mini kit (Qiagen).

All isolated RNA samples were quantified by the NanoDrop 2000 instrument (Thermo Fisher) and the RNA integrity was characterized by an RNA Quality Number (RQN) using the Fragment Analyzer capillary electrophoresis system (AATI) and Standard RNA kit (Agilent; tissue samples RQN = 9.2; range 5–10). Samples with RQN < 7.5 and c < 25 ng/µl of total RNA were excluded from further analysis.

Prior to the single strand cDNA synthesis, 3.75 µg of RNA of each sample was treated by DNase I (Thermo Fisher) according to the manufacturer’s instructions. Reverse transcription was performed in a 40 µl reaction using SuperScript III Reverse Transcriptase (Thermo Fisher) with random hexamers (Roche) as described previously^26^. All cDNA samples were stored in − 20 °C until the quantification step (performed within 1 month).

### Analysis of the overall *HNF1B* expression and *HNF1B* alternative splicing variants expression

The overall *HNF1B* expression analysis of cDNAs was performed by droplet digital PCR (ddPCR) approach using QX200 ddPCR system (Bio-Rad; including Automated Droplet Generator instrument) and EvaGreen quantification kits (Bio-Rad) as described by Dundr et al*.*^17^.

Several thorough optimization steps were performed prior to the analyses. These included the testing of expression of three pre-selected reference gene mRNA targets (*POLR2A*, *HPRT1*, and *ATP5F1B*; Table S1) and two *HNF1B* mRNA targets (in the 5′ UTR and 3′ UTR; Table S1), confirming the repeatability, reproducibility, optimal primers annealing temperature, and specificity (by Sanger sequencing). The reactions were prepared using QX200 ddPCR EvaGreen Supermix (Bio-Rad; according to the manufacturer’s instructions), 1 μl of cDNA template (which corresponded to approximately 90 ng of the total RNA), and 4 pmol of each of the primer pairs (200 nM final concentration) in 20 μl reaction volume.

The *HNF1B* ASVs expression analysis was performed by the same ddPCR system using probe kits as well as custom-designed FAM/HEX probes specific for unique and canonical exon-exon connections in one reaction for the direct evaluation of the alternative transcript amounts. Based on our previous research^19^, six major transcription variants of *HNF1B* were quantified (3p; Δ7; Δ7–8; Δ8; Δ5–8; and Δ6–8). For each variant, a pair of primers and probes were designed (Table S1). For example, the reaction which represents the Δ7 variant included the forward primer in exon 6 and the reverse primer in exon 8, the FAM probe specific for alternative exon 6 and exon 8 junction, and the HEX probe specific for canonical exon 6 and exon 7 junction. The optimization steps were performed similarly to the process described above, with the addition of a probe multiplexing step for each reaction. The reactions were prepared using ddPCR Supermix for Probes (No dUTP; Bio-Rad; according to the manufacturer’s instructions), 1 µl of cDNA template (approx. 90 ng of total RNA), 5 pmol of each of the two primers and two probes (250 nM final concentration) in a 20 µl reaction volume. Droplets were generated in the QX200 AutoDG instrument (Bio-Rad) according to the general instructions and amplified according to the manufacturer’s protocol (10 min incubation at 95 °C followed by 40 cycles of 94 °C for 20 s, 58 °C for 45 min, and final 98 °C for 10 min).

Data was acquired by the QX200 Droplet Reader instrument (Bio-Rad), using the standard acquisition protocol for Eva-Green/Probe master mix and analysed by the QuantaSoft software (Bio-Rad). The threshold for positive droplet signals of each of the individual amplicons was set as the average of the thresholds which were calculated automatically by QuantaSoft software during the optimization steps (48 reactions for each amplicon). The thresholds of all the acquired targets were manually confirmed. The final data of targets, expressed as the number of templates in 20 μl of master mix (which corresponded to 1 μl of cDNA), was recalculated in the following manner: (i) as the number of targets per reference *POLR2A* (overall *HNF1B* mRNA expression); (ii) as the percentage of the overall *HNF1B* variants (ASV relative expression; overall *HNF1B* expression was calculated as the sum of the full-length exon 3 variant and the 3p variant). The overall *HNF1B* expression was determined as the sum of the full-length exon 3 variant and the alternatively spliced 3p variant, which correlates to the overall *HNF1B* expression determined as the number of templates measured by the amplicon in 5′ UTR. The final recalculated data was analysed as described in the Statistics section.

The samples with *POLR2A* expression < 50 templates in 1 µl of cDNA were excluded from further statistical analysis of the overall *HNF1B* expression. The mean of the *POLR2A* expression in the whole dataset was 999 templates per 1 µl of cDNA (min = 58; max = 2076).

The samples with combined alternative exon 3 and canonical exon 3 expression > 20 templates/1 µl cDNA were evaluated as samples with sufficient *HNF1B* expression for further statistical analysis of individual ASVs expression. The mean of the *HNF1B* combined exon 3 and 3p expression in the subset was 141 templates per 1 µl of cDNA (min = 23; max = 3868).

### Analysis of ASV’s combination

Complementary DNA (cDNA) for the analysis of the potential combination of splicing events was synthesized using eight non-tumour RNA samples from the kidney as a template. The samples were equimolarly pooled into a mixture of 4 µg of total RNA, which was treated by DNase I, prior to cDNA synthesis as described in Hojny et al*.*^19^. The reverse transcription was performed according to the manufacturer’s protocol of SuperScript III Reverse Transcriptase (Thermo Fisher) in 40 µl reaction volume by using 1 µl of oligo dT primer (final concentration 1.25 µM). The created cDNA was amplified using forward primer in exon 2 and one of three reverse primers, which were specific for the unique exon-exon junctions of each of Δ7, Δ7–8, and Δ8 variant (the final concentration of primers was 200 nM; Table S1). The PCR reactions were mixed into 10 µl by using 5 × HOT FIREPol EvaGreen HRM Mix and amplified according to the instructions (95 °C—12 min; 35 cycles of 95 °C—15 s, 62 °C—20 s, 72 °C—1 min). The samples were visualized by 1% agarose gel electrophoresis. The PCR products were sequenced and confirmed by the direct Sanger sequencing.

### Statistical analyses

All statistical analyses were performed using the software Statistica v.13.5.0 (TIBCO Statistica) and/or the R software v.4.0.2^27^. The package “ggplot2”, implemented in R software, was used for data visualisation (violin plots, Figs. 2, 4). The Shapiro–Wilk test was used to control data normality. The non-parametric ANOVA approach (Mann–Whitney *U* test) was used to evaluate the associations between the *HNF1B* expression (overall or ASVs’) and the type of tissue (tumour vs non-tumour). For the pairwise comparisons (matched T and NT samples), Wilcoxon matched-pairs signed-ranks test was performed. The HNF1B protein expression (H-score) and RNA expression were correlated using Pearson’s method. All tests were two-sided and a p-value of less than 0.05 was considered as significant. All summary values were expressed as median.

The sample set from the pancreas was excluded from the statistical evaluation due to the insufficient number of samples suitable for analysis (10 T; 8 NT).

### Capture RNA-Seq library preparation, sequencing, and data analysis

The representative paired T and NT samples of RNA from six different tissues (pancreas, large intestine, prostate, kidney, EEC with paired tissue samples, and HGSC with paired tissue samples) were prepared similarly as described by Walker et al*.*^28^ using the standard KAPA RNA HyperPrep kit, enriched by the SeqCap protocol (Roche) by using a 257 kbp panel of gene targets (NimbleGen, Roche)^17^. The data was analysed by CLC Genomics Workbench v.21.0.3. software (Qiagen) by a standard RNA analysis pipeline. The mapped data was manually analysed in IGV (Broad Institute)^29^, and the identified splicing reads (reads which started in one exon and ended in another) were recalculated in the same way as the data from ddPCR—as a percentage of the overall *HNF1B* expression (overall *HNF1B* expression was calculated as the sum of splicing reads of the full-length exon 3 variant and the 3p variant).

### TCGA SpliceSeq data

The TCGA SpliceSeq^20^ (https://bioinformatics.mdanderson.org/TCGASpliceSeq) *HNF1B* data (based on whole transcriptome RNA-Seq approach) from colon adenocarcinoma (COAD), kidney renal clear cell carcinoma (KIRC) and prostate adenocarcinoma (PRAD) datasets together with their paired NT tissue samples was downloaded directly from TCGAss download page. Non-paired T samples and samples without detected ASV 3p expression (which is considered ubiquitous) were filtered out. The PSI values (percent splice in; percent of transcripts where the exon or variant is present) were recalculated as 100% – PSI which corresponds to a percentage where the variant is missing. Altogether paired T and NT samples of large intestine (COAD; N = 33); kidney (KIRC; N = 57) and prostate (PRAD; N = 38) datasets were further analysed. Downloaded data lacked variant Δ7, as a single exon skipping between exons 6 and 8. This variant was detected only in fraction of TCGA samples as the web viewer showed. In TCGAss, another variant 7 is present, but its expression is related to the exon 6–9 splice junction as skipped exon 7 together with skipped exon 7–8 variant. This calculation is not compliant with our approach and therefore we skipped this variant for further comparison.

### Database information

The information about the *HNF1B* transcripts was obtained from the RefSeq database as of April 30, 2021, where the spectrum of the fully characterized and predicted *HNF1B* transcripts was wider in comparison to the Ensembl database. The general information about the *HNF1B* mRNA expression in a variety of human tissues, as well as other used reference genes for mRNA expression, was obtained from the GTEx Portal database^7^.

## Supplementary Information

Supplementary Information.

## Data Availability

The data generated or analysed in this current study is included in this article (and its Supplementary Information Files). The raw data supporting the conclusions of this manuscript will be available from the corresponding author upon reasonable request to any qualified researcher.

## Abbreviations

CRC: Colorectal carcinoma
ccRCC: Clear cell renal carcinoma
PC: Prostate carcinoma
HGSC: High-grade serous carcinoma
T: Tumour
NT: Non-tumour
p: Deletion/intronization of 5′ exon part
Δ: Deletion/intronization of whole exon
ASV: Alternative splicing variant
NGS: Next-generation sequencing
RNA-Seq: RNA next-generation sequencing
ddPCR: Droplet digital PCR
RQN: RNA quality number
