# Supplementary material for: Comprehensive quantitative analysis of alternative splicing variants reveals the HNF1B mRNA splicing pattern in various tumour and non-tumour tissues
Source: Sci Rep. 2022 Jan 7;12:199. doi: 10.1038/s41598-021-03989-z (PMC8741901; doi:10.1038/s41598-021-03989-z)
Supplement: Supplementary file 1 — Supplementary Information. [file 41598_2021_3989_MOESM1_ESM.pdf]

Supplementary information

Comprehensive quantitative analysis of alternative splicing variants reveals the *HNF1B* mRNA splicing pattern in various tumour and non-tumour tissues

Jan Hojny[1], Romana Michalkova[1], Eva Krkavcova[1], Quang Hiep Bui[1], Michaela Bartu[1], Kristyna Nemejcova[1], Marta Kalousova[2], Petra Kleiblova[3], Pavel Dundr[1] and Ivana Struzinska[1*]

[1] Institute of Pathology, First Faculty of Medicine, Charles University and General University Hospital in Prague, Studničkova 2, 12800 Prague 2, Czech Republic. jan.hojny@vfn.cz (J.H.); romana.michalkova@vfn.cz (R.M.); eva.krkavcova@vfn.cz (E.K.); 105280@vfn.cz (Q.H.B.); michaela.bartu@vfn.cz (M.B.); kristyna.nemejcova@vfn.cz (K.N.); pavel.dundr@vfn.cz (P.D.); ivana.struzinska@vfn.cz (I.S.)

[2] Institute of Medical Biochemistry and Laboratory Diagnostics, First Faculty of Medicine, Charles University and General University Hospital in Prague, Kateřinská 32, 121 08 Prague 2, Czech Republic marta.kalousova@lf1.cuni.cz (M.K.)

[3] Institute of Biology and Medical Genetics, First Faculty of Medicine, Charles University and General University Hospital in Prague, Albertov 4, 128 00 Prague 2, Czech Republic petra.kleiblova@lf1.cuni.cz (P.K.)

[*] Correspondence:

ivana.struzinska@vfn.cz (I.S.)

Tel: +420224968685

Fax: +420224968635

Ivana Struzinska, Ph.D., Institute of Pathology, First Faculty of Medicine, Charles University and General University Hospital in Prague, Studničkova 2, 12800, Czech Republic

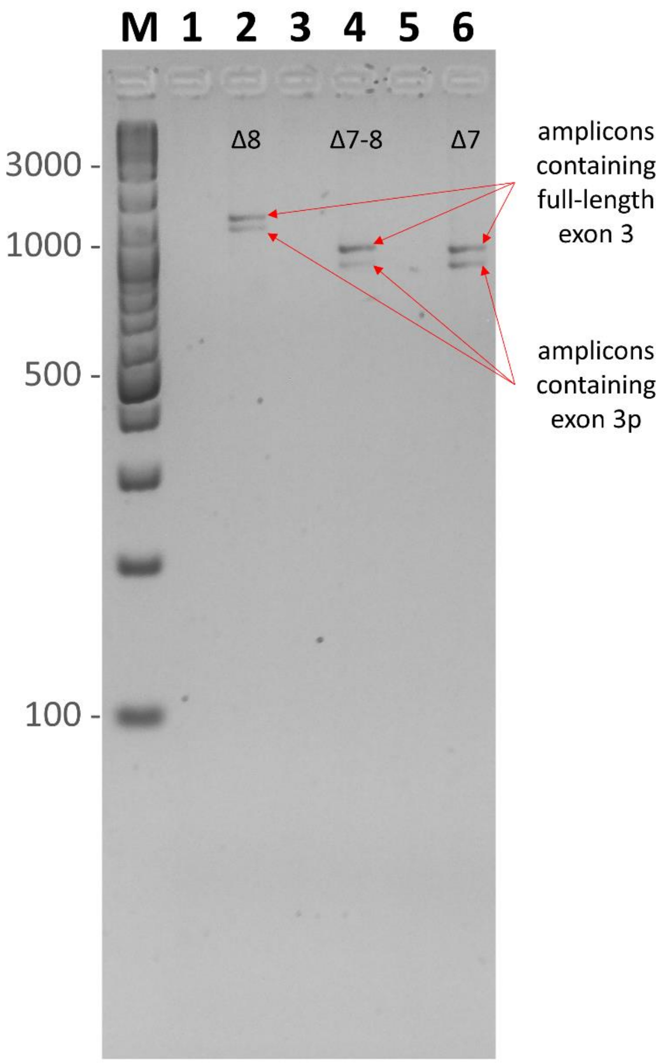

Figure S1. *HNF1B* ASVs Δ7, Δ7-8 and Δ8 exists in combination with the full-length exon 3 as well as the 3p ASV in the non-tumour kidney sample pool. cDNA sample pool was created by equimolar mixing of eight non-tumour kidney samples and amplified using combination of forward and reverse primer (Table S1c). The forward primer was in all reactions located in exon 2 and the reverse primer was located into unique exon 7 and exon 9 junction (lane 2: Δ8: 1099 bp full-length exon 3; 1021 bp 3p ASV); unique exon 6 and exon 9 junction (lane 4: Δ7-8; 904 bp full-length exon 3; 826 bp 3p ASV); or unique exon 6 and exon 8 junction (lane 6: Δ7: 901 bp full-length exon 3; 823 bp 3p ASV). Two products were detected in all primer mixes (lanes 2,4 and 6). Longer product corresponds to the amplicons containing the full-length exon 3 as indicated by red arrows, while the shorter product corresponds to the amplicon containing 3p ASV as indicated by red arrows. Lanes 1, 3, and 5 represents respective negative control reactions. M – 100 bp ladder. PCR amplicons are visualized by UV-light after electrophoretic separation in 1% agarose gel. The photo of the gel was cropped from the surroundings, transformed to negative and grey colour scheme and overturned. The original photo of the gel is below.

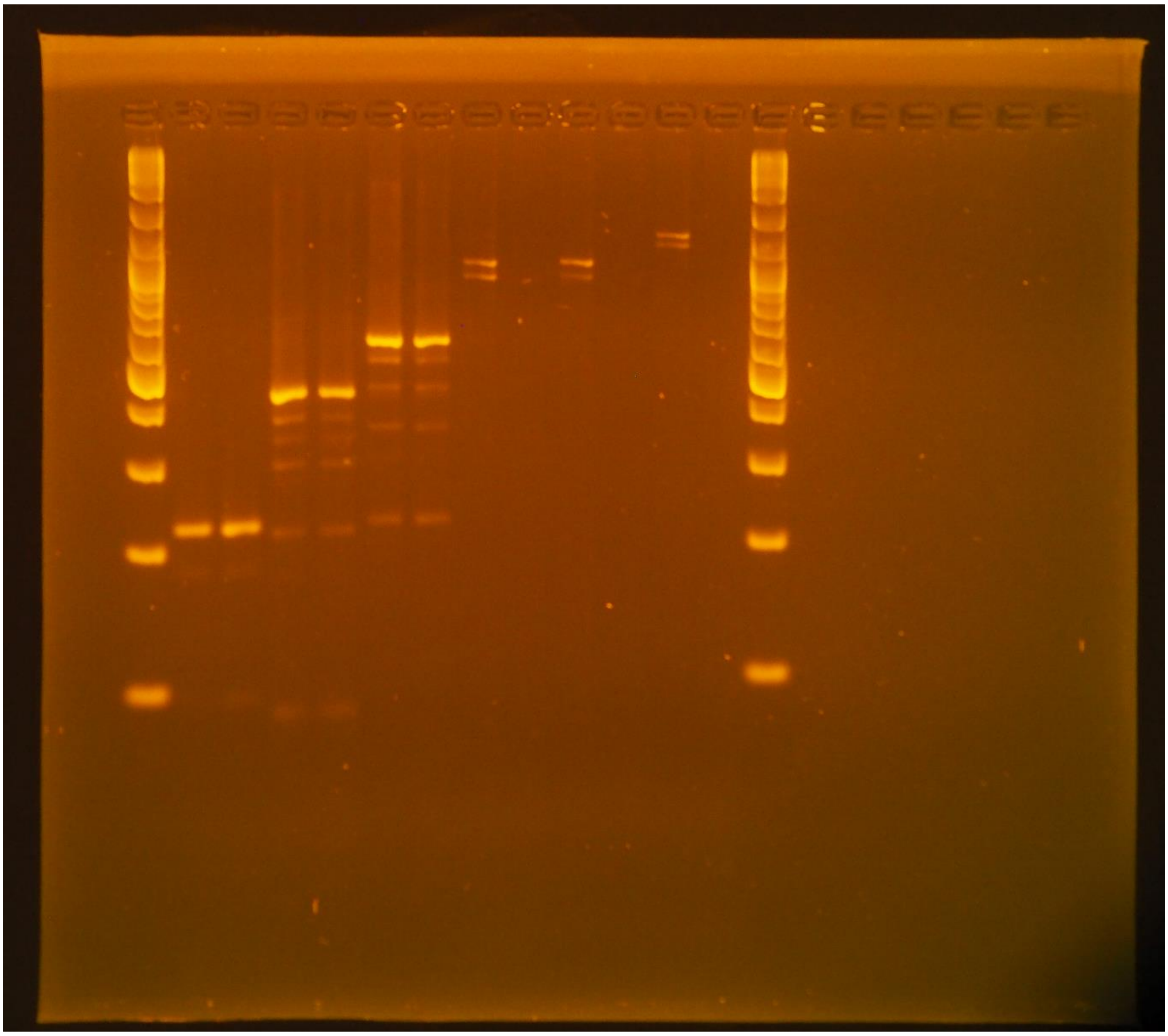

Original photo of the agarose gel electrophoresis.

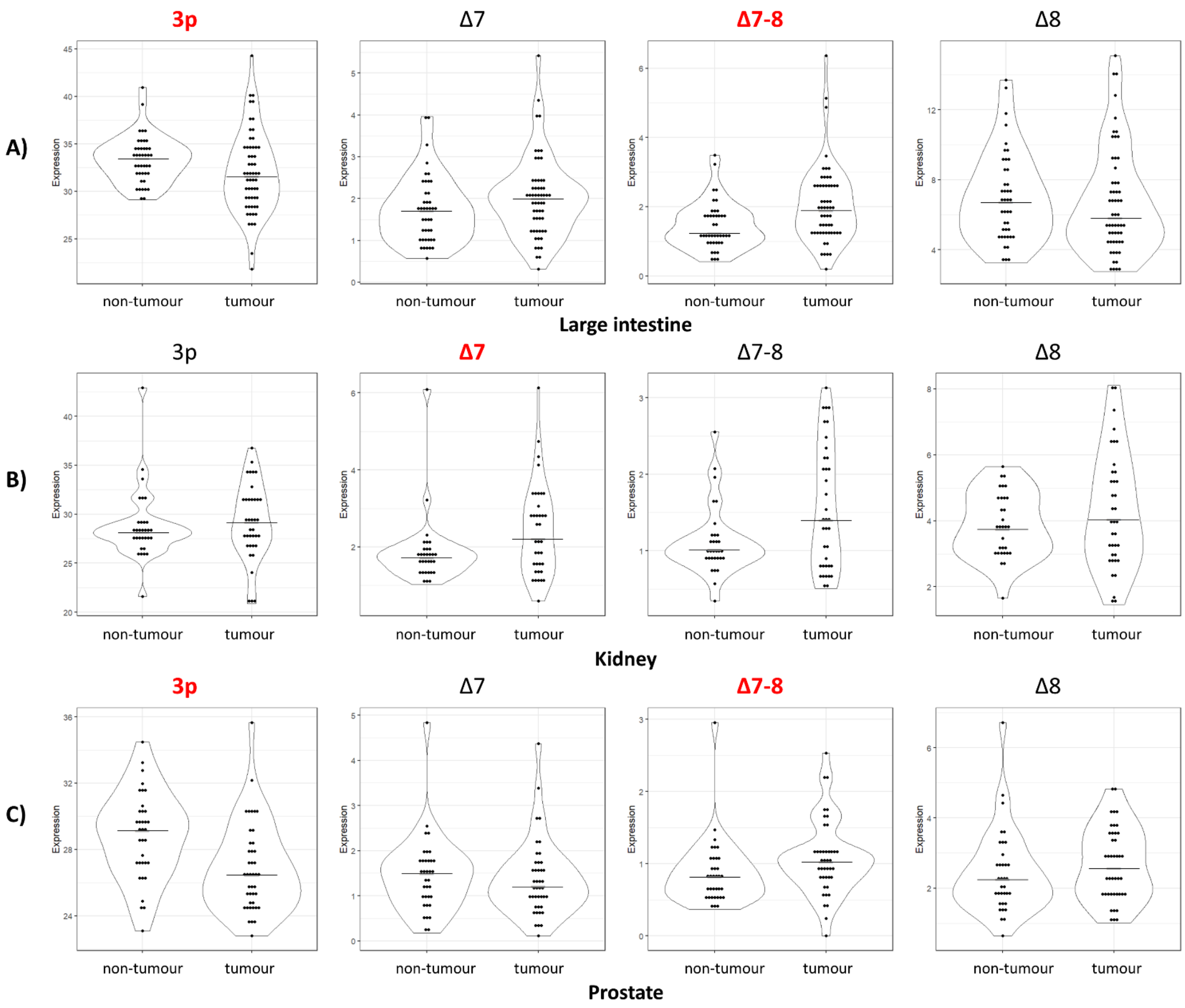

Figure S2. Expression levels of all analysed *HNF1B* alternative splicing variants in NT and T tissue sample sets. **A)** large intestine (NT = 42 samples; T = 57 samples); **B)** kidney (NT = 31 samples; T = 37 samples); **C)** prostate (NT = 35 samples; T = 42 samples). Data is visualized as violin plots. Each dot represents one sample. Expression is relative to overall *HNF1B* mRNA expression (100). Black line represents median. Red names of the ASVs indicates significantly different expression between NT and T sample in particular tissue type.

Table S1. List of primer and probe sequences

A) Primer pairs used for overall *HNF1B* quantification by ddPCR				
Name	Forward primer (5' - 3')	Tan (°C)	Reverse primer (5' - 3')	Tan (°C)
HNF1B 5UTR	CATGGCAAGTTAGAAGTTTTCTGACTCC	58.1	GGAGTCAGAAAACTTCTAACTTGCCATG	58.4
HNF1B 3UTR	CTGCTGGCACCTCAGACAATC	57.6	CAAGGACTCCTGTCTGTCCTGG	58.1
POLR2A	GCATGTTCTTTGGTTCAGCACC	57.1	GAGTGGAATGACCCCAGGGG	58.2
HPRT1	GTGATGATGAACCAGGTTATGACCTTG	57.7	CGTCTTGCTCGAGATGTGATGAAG	57.8
ATP5F1B	GCTCCCATTCATGCTGAGGC	57.9	GGCTTTTTGGTGGTGCTGGA	57.8

B) Primer pairs and probes used for ASV's quantification by ddPCR									
Name	Forward primer (5' - 3')	Tan (°C)	Reverse primer (5' - 3')	Tan (°C)	Junction	Probe sequence (5' - 3')	Tm (°C)	GC (%)	Dye
HNF1B 3p	GCTCTGTACACCTGGTACGTC	56.5	CATCATCGGACTGCCCAGG	56.8	alternative	AGATCCTCCGACAGTTCAGTCAAC	58	50	Fam
					canonical	AGATCCTCCGACAATTCAACCAGAC	58.4	48	Hex
HNF1B del5-8	CACCACCAGCCCAGCTC	56.9	GCACGAAGTAAGTGGTGTGTG	56.1	alternative	AACAAGCTGTCAGTGTCCTCTACAAG	58.6	46.2	Fam
					canonical	CAAGCTGTCAGGAGTGCGCT	58.3	63.2	Hex
HNF1B del6-8	GACCCAGGCCACAATCTCC	56.6	GCACGAAGTAAGTGGTGTGTG	56.1	alternative	CTGATGGTAAAATGTGTCCTCTACAAGC	57.8	42.9	Fam
					canonical	CTGATGGTAAAATGATCTCAGTCTCAGGA	57.8	41.2	Hex
HNF1B del7	CATCATGACACCCCTCTCTGG	56.1	GGGAGGTGTGGGAATACTGGG	58	alternative	CAATTGCACAAATGTACGCACACAAG	58.5	42.3	Fam
					canonical	CAATTGCACAAAGCCTCAACACCT	58.7	45.8	Hex
HNF1B del7-8	CATCATGACACCCCTCTCTGG	56.1	GCACGAAGTAAGTGGTGTGTG	56.1	alternative	TGGCAATTGCACAAATGTCCTCTAC	58.1	44	Fam
					canonical	CAATTGCACAAAGCCTCAACACC	57.7	47.8	Hex
HNF1B del8	CCAGCAGCCCTTCATGGC	57.8	GCACGAAGTAAGTGGTGTGTG	56.1	alternative	CAGAACTCACACATGTCCTCTACAAGC	59.2	48.1	Fam
					canonical	CAGAACTCACACATGTACGCACACA	59.6	48	Hex

C) Primer pairs used for analysis of ASV's combination				
Name	Forward primer (5' - 3')	Tan (°C)	Reverse primer (5' - 3')	Tan (°C)
HNF1B del7	GCTCTGTACACCTGGTACGTC	56.5	GCACAAATGTACGCACACAAGC	58
HNF1B del7-8	GCTCTGTACACCTGGTACGTC	56.5	GCACAAATGTCCTCTACAAGCCTG	58.1
HNF1B del8	GCTCTGTACACCTGGTACGTC	56.5	CTCACACATGTCCTCTACAAGCCT	58

Tan (°C) represents annealing temperature of particular primer or probe calculated by AnnHyb v4.946 (http://bioinformatics.org/annhyb); GC (%) represents percentage of G and C nucleotides in designed probe sequence; Dye represents type of used fluorophore of the quencher probe.
